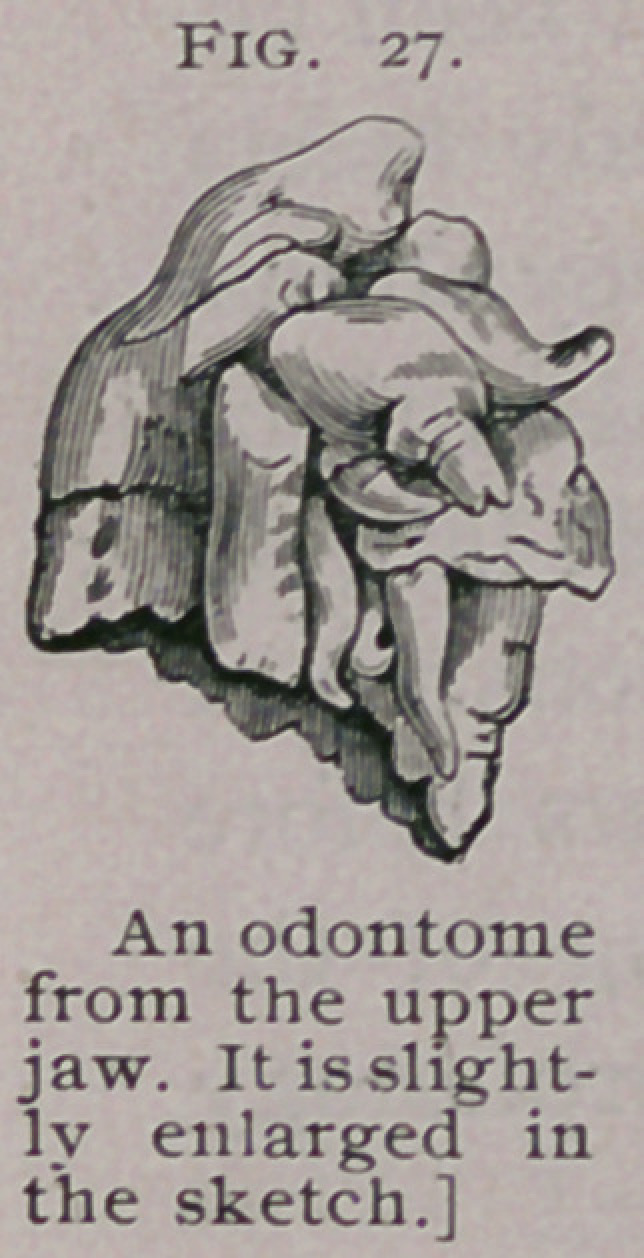# Odontomes

**Published:** 1890-02

**Authors:** J. Bland Sutton

**Affiliations:** Assistant Surgeon, Middlesex Hospital, London; Erasmus Wilson Lecturer, Royal College of Surgeons, England


THE JOURNAL
OF
COMPARATIVE MEDICINE AND
VETERINARY ARCHIVES.

VOL. XI. FEBRUARY 1890. No. 2.

### ODONTOMES.

By J. BLAND SUTTON, F.R.C.S.,
*Assistant Surgeon, Middlesex Hospital, London ; Erasmus Wilson Lecturer, Royal College of Surgeons, England.*

[*Continued from page 11.*]

*Radicular Odontomes.*—This term is applied to odontomes which arise after the crown of the tooth has been completed, and while the roots are in the process of formation. As the crown of the tooth, when once formed, is unalterable, it naturally follows that should the root develop an odontome, enamel cannot enter into its composition, which, for the most part, would consist of dentine and osteo-dentine in varying proportions, these two tissues being the result of the activity of the papilla.

As a typical radicular odontome, we may choose the well-known specimen described by Salter, and represented on next page Fig. 15. In this specimen the tumor is clearly connected with the fangs. The outer layer of the odontome is composed of cementum ; within this is a layer of dentine, deficient in the lower part of the tumor ; within this is a nucleus of calcified pulp.

FIG. 15.

A radicular odontome from the human subject. *A* represents the natural size of the specimen. [After Salter].

Mr. Hare, of Limerick, removed from the upper jaw of a man aged 41 the odontome sketched in Fig. 16. This specimen was originally described by Sir John Tomes[1] in 1863, but it was examined and redescribed in 1872, by Mr. Charles Tomes.[2] The mass is invested by cementum; inside this casing is a shell of dentine, the tubes radiating outward and disposed with some regularity. This dentine was deficient at the distal end of the tumor; its interior was filled with an ill-defined osseous material.

FIG. 16.

A radicular odontome. [After John Tomes.]

Radicular odontomes are rare in man, but frequent in other mammals, and are often multiple. Rodents are especially liable to them. In a Quebec marmot I found an odontome connected with the root of each upper incisor. The tumors had produced absorption of the hard palate and projected into the mouth. In a young marmot I met with four odontomes, one attached to each incisor in the upper and lower jaw. One of them is sketched *in situ*, Fig. 17, and of natural size. It con-

[1] *Trans. Odont. Soc. of Great Britain*, 1863.
[2] *Trans. Odont. Soc. of Great Britain*, 1872.

sisted mainly of cementum. A similar tumor came under my notice in a Canadian porcupine. The odontome is shown of natural size in Fig. 18. It consisted mainly of dentine. The tumor was lodged in a large pus-containing cavity, and the surrounding bone was bare and dead. I have recorded a similar specimen in an agouti. In all these cases death was probably due to the profuse suppuration set up by the odontomes, the pus being drawn into the air passages, setting up septic pneumonia.

Fig. 17.

The left lower jaw of a young marmot with a large radicular odontome connected with the incisor.

Radicular odontomes have been obtained from elephants, arising in connection with the roots of the tusks; indeed the largest odontomes on record were obtained from elephants. The Museum of the Royal College of Surgeons contains several excellent specimens. Structurally they consist almost entirely of osteo-dentine. A radicular odontome described by Windle & Humphreys is represented in Fig. 19. It was obtained from a man 25 years of age.

Fig. 18.

The lower jaw of an adult Canadian porcupine. A radicular odontome is attached to its lower incisor.

The odontome was situated in the lower jaw, on the right side, in the neighborhood of the second molar tooth. After more than four months excruciating pain, accompanied with profuse suppuration, life being several times

Fig. 19.

Two drawings of a radicular cementoma, from a man aged 25 years.

despaired of, the odontome, seven months after its presence was first noticed, became liberated and fell into his mouth. The tumor is represented of natural size in the figures. The crown is fairly well formed, the labial surface being perfect, the lingual somewhat tuberculated. The roots are fused into an irregular mass. The under surface is irregular, and is at one point excavated into a hollow. It is much to be regretted that it is impossible to obtain sections of this interesting tumor.

*C.* ABERRATIONS OF THE WHOLE TOOTH-GERM.

*Composite Odontomata.*—This is a convenient term to apply to those hard tooth tumors which bear little or no resemblance in shape to teeth, but occur in the jaws, consisting of a disordered conglomeration of enamel, dentine, and cementum. Such odontomes may be considered as arising from an abnormal growth of all the elements of a tooth-germ—enamel-organ, papilla, and follicle.

Not only is this class of odontomes composite in that the tumors comprised in it originate from all the elements of a tooth-germ, but they are composite in another sense. In the majority of cases the tumor is composed of two or more tooth-germs indiscriminately fused. But they differ from the cementomata containing two or more teeth from the fact that the various parts of the teeth composing the mass are indistinguishably mixed, whereas the individual teeth implicated in a cementoma can be clearly defined.

Up to the present time I have found no such odontomes in the lower animals, all the recorded cases having occurred in man. A typical odontome of this group is the one described by Mr. Heath[1] as occurring in the lower jaw of a young lady aged 18. The clinical history in this case is very instructive, and the reader is referred to the original account of it. Fig. 20.

FIG. 20.

A composite odontome from a young lady aged 18. [After Heath.]

The specimen is further valuable on account of the exhaustive and careful histological examination made by Mr. Charles Tomes, who found it composed of enamel, dentine and osteo-dentine.

The museum of the Middlesex

[1] Clinical Society's Transactions, vol. xv.

Hospital contains an odontome, but unfortunately it is without history and nothing can be ascertained concerning it except the specimen came from the human subject. The odontome was presented to the collection by Mr. Pearce Gould. It is represented of natural size in Fig. 21, and weighs 160 grains.

I have examined the tumor histologically. It is scroll-like in form, having its convex surface dotted with minute projecting denticles. It is composed of cementum largely mixed with dentine. The denticles are entirely composed of this substance, and on section are seen to be deeply embedded in the mass of the tumor. Enamel is present in very small quantity.

FIG. 21.

A complete odontome. [Mr. Pearce Gould's case.]

Forget's well-known case belongs to this class. The patient was aged 20, but the disease had been noticed since the age of 5 years. Behind the first bicuspid no teeth were seen, but the jaw as far back as the ramus was the seat of a smooth, unyielding tumor. The parts represented in the figure were removed during life.

FIG. 22.

A composite odontome *in situ*. [After Forget.]

On microscopic examination the tumor was found to consist mainly of dentine, the surface of which was in places covered with enamel dipping down into the crevices, at the bottom of which cementum was found.

The Transactions of the Pathological Society, London, though a mine of wealth in most kinds of tumors, contain only one description of an odontome. It is described in Vol. xxxii, by Mr. Rushton Parker. The specimen originated in connection with the second left lower molar of a lady aged 19 years. An attempt was made to extract the tooth, but it broke, leaving the tumor behind. Subsequently an attempt made to extract the mass failed, a few fragments only being detached; about two years later it issued spontaneously from the alveolus. The odontome, which weighs 136 grains, is represented of natural size in Fig. 23, taken from a drawing kindly furnished me by Mr. Rushton Parker. A cast of the specimen is preserved in the museum of Middlesex Hospital.

In the specimen removed by Sir W. Fergusson, the tumor consisted of dentine, cementum and enamel thrown irregularly together. As in the preceding case, a large portion of the jaw was removed with the odontome, the true nature of the tumor not being recognized before the operation.

In the same category may be placed the odontome dislodged by Professor Annandale[1] from the lower jaw of a girl aged 17. It weighed 300 grains. It consisted of dentine and osteodentine, capped by enamel.

FIG. 23.

An odontome. (Mr. Rushton Parker's case.)]

Nine months before the patient was seen by Mr. Annandale, an abscess formed over the top, from which the odontome was ultimately dislodged; the abscess left a chronic sinus from which small quantities of pus issued up to the time of the operation. No molar teeth were erupted in the right lower jaw, their position being occupied by the odontome. The cavity left by the dislodgement of the odontome was lined with a smooth, velvety membrane.

It is supposed that odontomes are more frequent in the lower than the upper jaw, but there is good ground for the belief that

[1] *Edin. Med. and Surg. Journal*, 1873.

many such tumors have been described as exostoses of the antrum. Thus M. Michon removed from the antrum of a Frenchman, aged 19 years, at the Hôpital de la Pitié, (without an anæsthetic), the large odontome represented in Fig. 24. The operation, which may be described as a "surgical struggle," lasted upwards of an hour and a quarter.

The tumor is described as an exostosis, but fortunately M. Michon's account is accompanied by some excellent drawings which show clearly enough that the tumor is an odontome. The cut surface exhibited a laminated disposition. Microscopically it

FIG. 24.

An odontome from the upper jaw, natural size. [M. Michon's case.]

was composed of tissue presenting many parallel tubules having the appearance of exaggerated dentinal tubes. It is the largest odontome from man of which we have any record; its weight is 1080 grains.[1] A case almost parallel with that of M. Michon has been described by Dr. T. Duka,[2] by whom it was removed from a Mahomedan woman, aged 26 years, at Monghyr, Bengal. The woman had for six years suffered from a muco-purulent discharge from the right nostril, and was now anxious for relief.

[1] For details of the case see *Mem. de la Société de la Chir., Paris*, 1850.

[2] *Trans. Path. Soc.*, Vol. xvii. A case of removal of a part of the superior maxillary bone on account of a bony tumor in the nasal fossa.

The case was regarded as one of necrosis, but after a "surgical struggle" lasting nearly an hour without chloroform, the tumor represented in Fig. 25 was withdrawn from the antrum. It had no connection with the surrounding tissues.

FIG. 25.

A composite odontome from the upper jaw, natural size. [Dr. Duka's case.]

The tumor, which was regarded as an exostosis, was submitted to a committee of the Pathological Society. In its report this committee states that the bone tissue differs in character from that ordinarily seen in exostoses. The tumor is preserved in St. George's Hospital Museum. An examination of the specimen, and an inspection of the drawings illustrating the above-mentioned report, show clearly enough that it is a composite odontome.

Dr. Duka in his account of the case states that Dr. Allen Webb was of opinion that the nucleus was formed by a tooth follicle escaping into the antrum of Highmore.

Mr. Jordan Lloyd[1] has published an excellent account of an odontome of this class which he removed from the right upper jaw of a young man. As so often happens, the case was regarded as one of necrosis, but when removed from its bed was recognized as an odontome.

The tumor is represented of natural size, entire and in section, Fig. 26. It weighs 279 grains. It is composed of osteo-dentine, with cementum here and there. Opaline, pearly patches are

1 *Lancet*, 1888.

studded irregularly around the edge of the cut surface. The mass occupied the space of the second, and probably the third, right upper molars ; it could be felt to be slightly loose before attempts were made to remove it. After its extraction a deep, round, smooth, velvet-like cavity remained, and the exposed part, with its crater-like hollow and surrounding ridge, bore a certain resemblance to a molar tooth crown.

FIG. 26.

A composite odontome from the upper jaw, natural size. The left hand figure shows the tumor in section. [Mr. Jordan Lloyd's case.]

The odontome represented in Fig. 27 was removed by Mr. S. Brock from a lad aged 19 years ; it was situated in front of the right upper bicuspid, displacing the lateral incisor and canine, so as to occupy their position in the dental arch. As will be seen in the sketch, it has no fang, and appears to consist merely of a crown and neck, but the crown bristles with cusps ; as many as nine distinct enamel-covered eminences can be detected.

FIG. 27.

An odontome from the upper jaw. It is slightly enlarged in the sketch.]

I have on several occasions pointed out that odontomes resemble teeth in this way—for a time during their development they remain hidden below the mucous membrane, and give no evidence (or very little) of their existence. To this succeeds an eruptive stage, and the suppuration, with the constitutional disturbance dependent thereon, draw attention to them. If this view be correct, then this remarkable structure must be regarded as an odontome which has cut the gum and taken a position in the dental series. This specimen is further interesting in that it consists of a conglomeration of denticles, for I have urged that those remarkable cases in which denticles have from time to time been erupted from a tumor connected with the jaw should be classed as odontomes. It is easy to imagine that if the cusps of this odontome remained distinct and each had been separately erupted, they would have been called supernumerary teeth. Indeed, many of the cusps can be easily detached from the main mass. Thus this strange specimen serves to bridge the gap between what I call compound follicular cysts and composite odontomes.

## Figures and Tables

**Fig. 15. f1:**
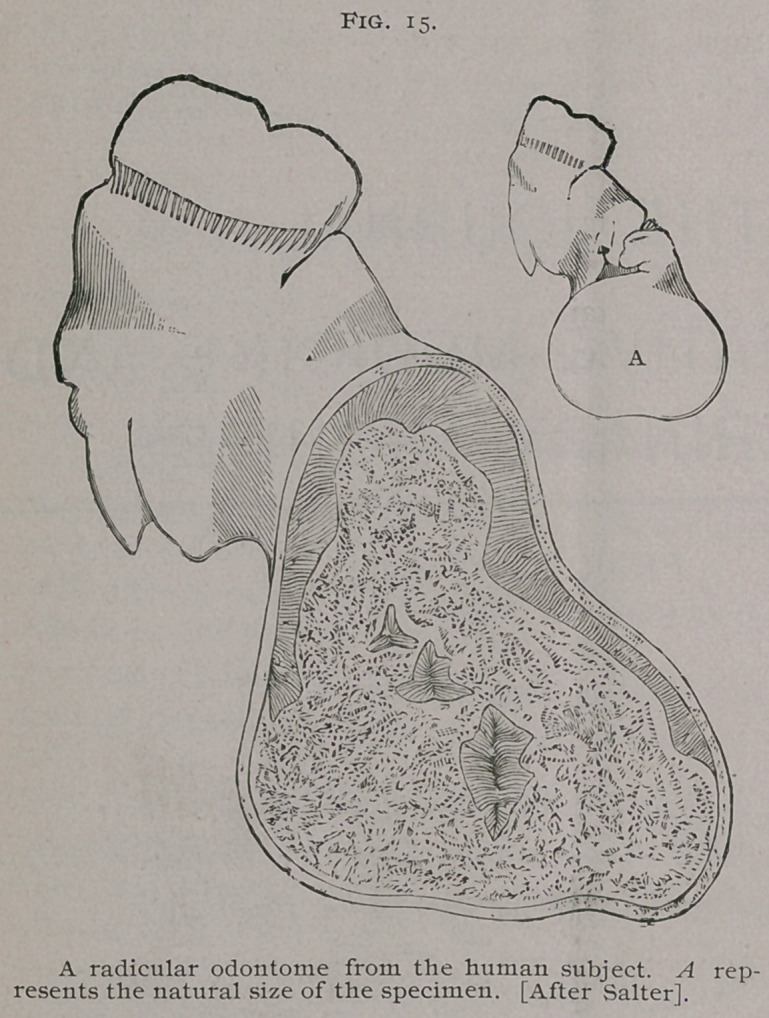


**Fig. 16. f2:**
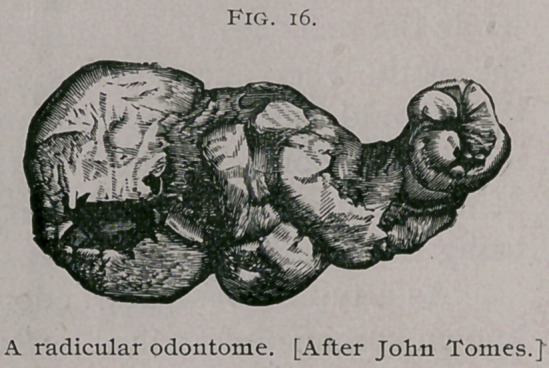


**Fig. 17. f3:**
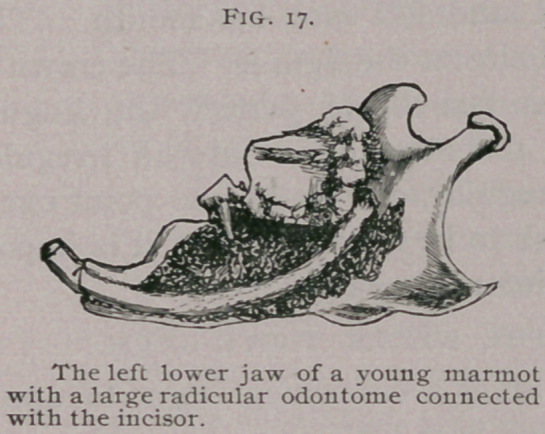


**Fig. 18. f4:**
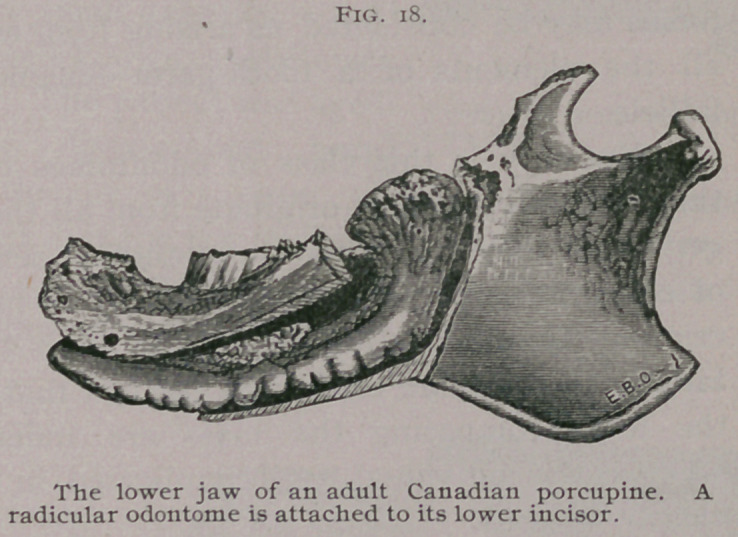


**Fig. 19. f5:**
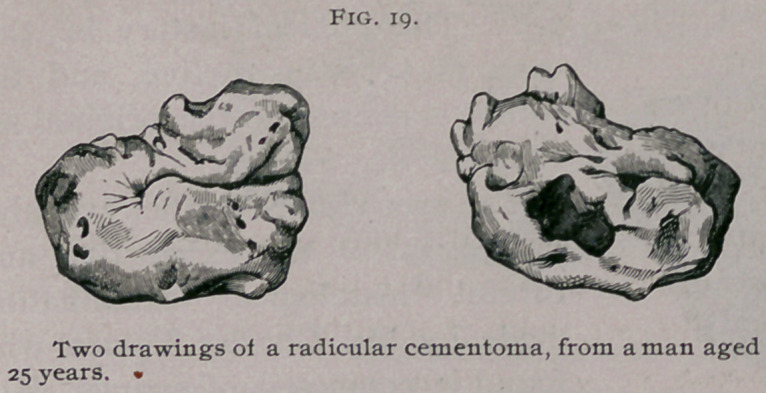


**Fig. 20. f6:**
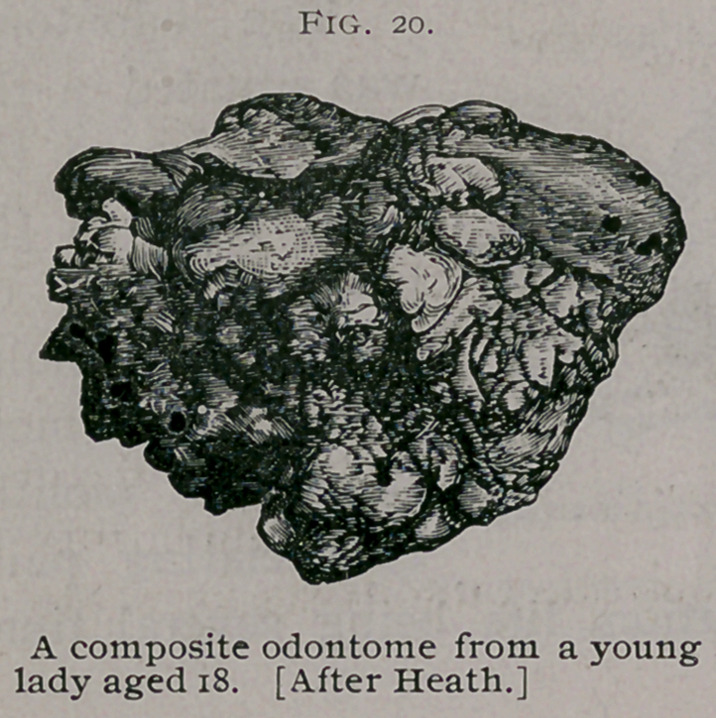


**Fig. 21. f7:**
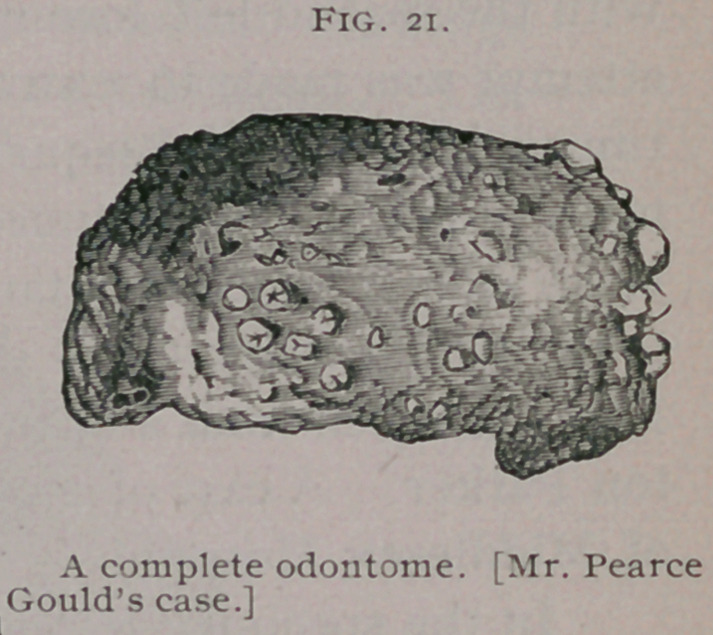


**Fig. 22. f8:**
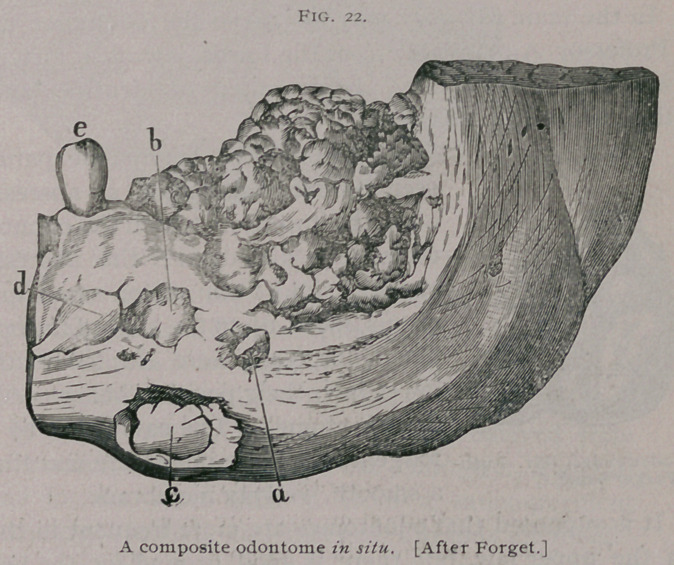


**Fig. 23. f9:**
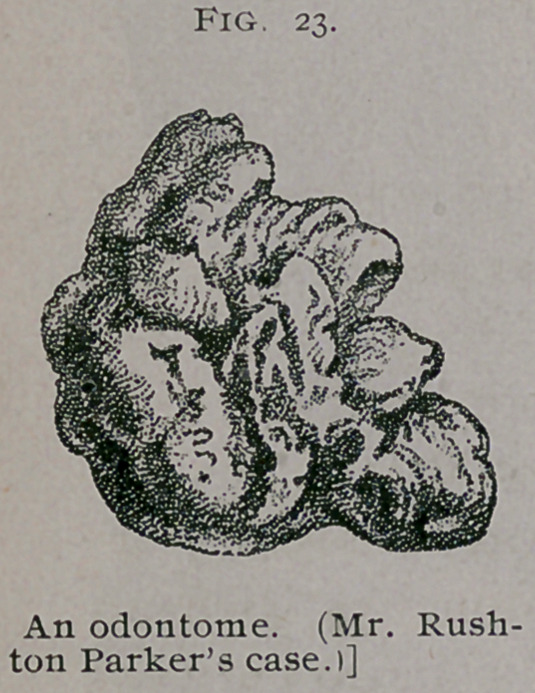


**Fig. 24. f10:**
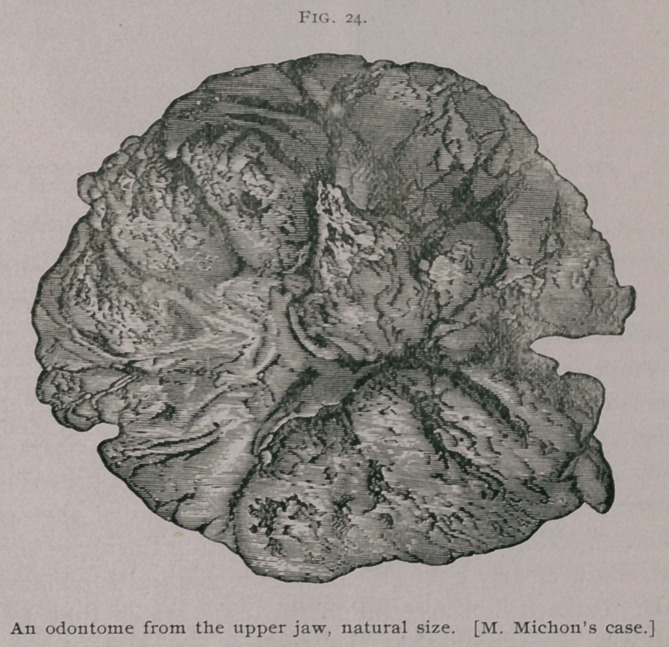


**Fig. 25. f11:**
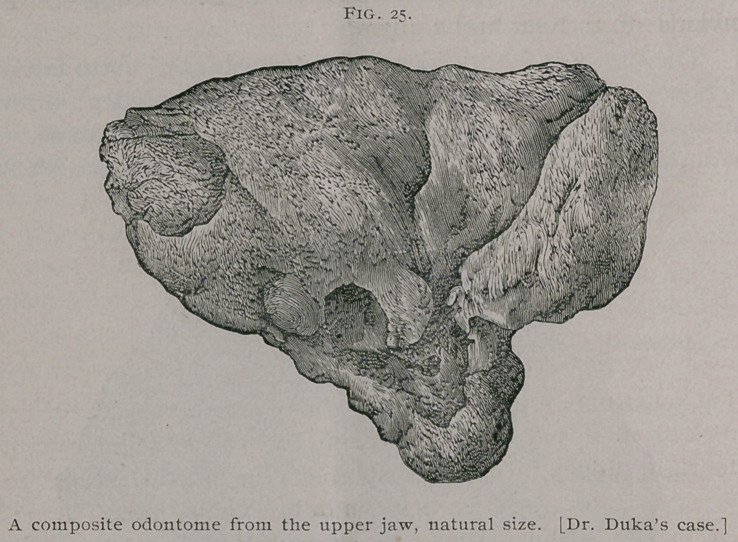


**Fig. 26. f12:**
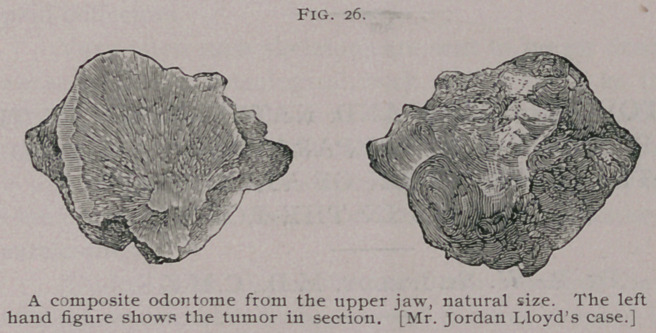


**Fig. 27. f13:**